# 1989. Surge in Group A Streptococcal Infections in England during 2022

**DOI:** 10.1093/ofid/ofad500.116

**Published:** 2023-11-27

**Authors:** Rebecca L Guy, Colin S Brown, Emily L Mason, Helen E Hughes, Alicia Demirjian, Mariyam Mirfenderesky, Sarah R Anderson, Derren Ready, Obaghe Edeghere, Theresa Lamagni

**Affiliations:** UK Health Security Agency, London, England, United Kingdom; UK Health Security Agency, London, England, United Kingdom; UK Health Security Agency, London, England, United Kingdom; UK Health Security Agency, London, England, United Kingdom; UK Health Security Agency, London, England, United Kingdom; UK Health Security Agency, London, England, United Kingdom; UK Health Security Agency, London, England, United Kingdom; UK Health Security Agency, London, England, United Kingdom; UK Health Security Agency, London, England, United Kingdom; UK Health Security Agency, London, England, United Kingdom

## Abstract

**Background:**

Following removal of non-pharmaceutical interventions (NPI) to restrict SARS-CoV-2 transmission in England, large increases above seasonally expected levels of group A streptococcal (GAS) infections and associated deaths were seen, particularly in children, during 2022.

**Methods:**

Nationwide data from UK Health Security Agency surveillance databases were extracted, including statutory clinical notifications of scarlet fever and laboratory-confirmed invasive GAS infections (iGAS; sterile-site specimens). Analyses compared infection and mortality rates in England for the pre-COVID-19 pandemic (2017-2019), pandemic-NPI (2020-2021), and post removal of COVID-19 NPI (post-NPI; 2022-2023) periods.

**Results:**

Pre-pandemic, a mean of 2481 iGAS cases (range 2138-2921; 13%-14% < 15y; Figure) per-year were recorded in England (4.4/100,000 population; 95% confidence interval (CI):4.3-4.6). Case numbers fell markedly during the pandemic-NPI period, 1462 iGAS cases in 2020 (2.6/100,000; 7% aged < 15y) and 829 in 2021 (1.5/100,000; 6% < 15y; Figure). Post-NPI cessation, iGAS cases increased to 2892 (5.1/10,000; 24% < 15y) in 2022, remaining high into Jan-Mar 2023 (1361; 21% < 15y). Scarlet fever diagnoses similarly increased, with 54,630 (91.6/100,000; CI: 90.8-92.4) notified in 2022 (England & Wales), the highest number since 1953.

In 2022, 339 deaths (< 7d iGAS diagnosis) were reported (case-fatality rate (CFR) 11.7%; CI:10.6-13.0%; 16% deaths were aged < 15y), compared to 214-335 per-year pre-pandemic (8%-10% in < 15y) and 88 in 2021 (6%< 15y). Respiratory virus co-infections were identified in 19% iGAS aged < 15y during 2022, CFR: 19.8%.

Post-NPI cessation, strain typing identified increasing dominance of *emm*1 and *emm*12 (all ages: 35% and 18%) in 2022, compared with 3% and 2% in pandemic-NPI and 23% and 6% pre-pandemic (all-ages).

**Figure:**
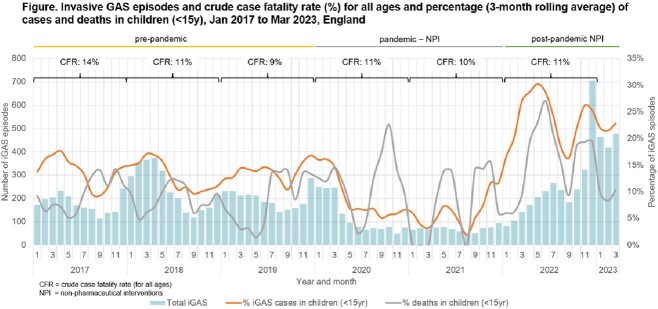

**Conclusion:**

iGAS presentations showed a marked rebound in 2022, most notably in children, with re-emergence of *emm*1. The rapid, steep increase in morbidity was possibly driven by increased opportunities for exposure and sub-optimal immunity following COVID-19 NPI.

**Disclosures:**

**All Authors**: No reported disclosures

